# A new understanding of coronary curvature and haemodynamic impact on the course of plaque onset and progression

**DOI:** 10.1098/rsos.241267

**Published:** 2024-09-11

**Authors:** Mingzi Zhang, Ramtin Gharleghi, Chi Shen, Susann Beier

**Affiliations:** ^1^ School of Mechanical and Manufacturing Engineering, University of New South Wales, Sydney, New South Wales 2052, Australia

**Keywords:** coronary artery disease, atherosclerosis, stenosis, anatomical characteristics, computational fluid dynamics

## Abstract

The strong link between atherosclerosis and luminal biomechanical stresses is well established. Yet, this understanding has not translated into preventative coronary diagnostic imaging, particularly due to the under-explored role of coronary anatomy and haemodynamics in plaque onset, which we aim to address with this work. The left coronary trees of 20 non-stenosed (%diameter stenosis [%DS] = 0), 12 moderately (0 < %DS < 70) and 7 severely (%DS ≥ 70) stenosed cases were dissected into bifurcating and non-bifurcating segments for whole-tree and segment-specific comparisons, correlating nine three-dimensional coronary anatomical features, topological shear variation index (TSVI) and luminal areas subject to low time-average endothelial shear stress (%LowTAESS), high oscillatory shear index (%HighOSI) and high relative residence time (%HighRRT). We found that TSVI is the only metric consistently differing between non-stenosed and stenosed cases across the whole tree, bifurcating and non-bifurcating segments (*p *< 0.002, AUC = 0.876), whereas average curvature and %HighOSI differed only for the whole trees (*p *< 0.024) and non-bifurcating segments (*p *< 0.027), with AUC > 0.711. Coronary trees with moderate or severe stenoses differed only in %LowTAESS (*p *= 0.009) and %HighRRT (*p *= 0.012). This suggests TSVI, curvature and %HighOSI are potential factors driving plaque onset, with greater predictive performance than the previously recognized %LowTAESS and %HighRRT, which appears to play a role in plaque progression.

## 1. Introduction

Coronary artery disease (CAD) primarily manifests as the buildup of atherosclerotic plaques in major epicardial arteries, which can lead to myocardial ischaemia and even death, responsible for much mortality and morbidity [1]. Consequently, early identification of patients at risk for coronary atherosclerosis may offer opportunities for prevention and early diagnosis of CAD. While the risk of CAD has been linked to factors such as age, sex, smoking history, cholesterol levels and blood pressure [2], there are still considerable variations among individuals in terms of the sites of plaque onset and the subsequent rate of plaque progression [3]. Together, nearly 25% of cardiovascular events remain unexplained by our current understanding [4], highlighting the need for further research into the residual risk factors [1].

In the past decades, coronary anatomy [5,6] and endothelial shear stress (ESS, or wall shear stress, WSS) [7–10] have been linked to atherosclerotic plaque development. Various prognostic metrics have been proposed, including coronary curvature, oscillatory shear stress [5,11], relative residence time (RRT) [12,13], and, more recently, WSS multidirectionality and topology [14–18]. Despite extensive research into their effects on plaque progression/vulnerability, their impact on the initial stage of plaque onset remains underexplored, largely due to the lack of longitudinal follow-ups starting from the healthy state.

For plaque progression, consistent findings across studies in humans and in pigs point low ESS to a greater increase in plaque burden [7,9,10], fibrous tissues [19] and progression rate [20]. However, the effects of high WSS remain inconclusive. For instance, high WSS has been linked to both regression of total plaque area and fibrous tissues [19] and progression of plaques towards a more vulnerable phenotype, predicting myocardial ischaemia [21]. Similarly, the role of oscillatory shear stress varies [19,20]. These inconsistent findings may be partly due to the different methods and thresholds used to calculate and classify adverse haemodynamics, as coronary branches exhibit distinct WSS magnitude and distributions [22], and thus a relative measure is needed.

For plaque onset, only a limited number of investigations exist, which were mainly retrospective or on animals [23,24]. Haemodynamics under the healthy state was usually assessed through computational fluid dynamics (CFD) performed on diseased coronaries with plaques virtually removed [24,25]. While these studies have highlighted the prognostic value of time-averaged ESS (TAESS), oscillatory shear index (OSI) and RRT, the manual plaque removal process could introduce uncertainties, potentially under- or over-estimating the size of the healthy lumen [26]. Regarding coronary anatomy, its governing effects on the intensity of helical flow [27] and the low ESS [5] have been extensively studied. However, its predictive value for plaque onset remains poorly understood, in contrast to their well-studied role in predicting plaque progression [6].

Leveraging an expert-annotated coronary artery dataset that previously served as a benchmark for the Automated Segmentation Of normal and diseased Coronary Arteries (ASOCA) challenge [28], we aim to compare the left coronary tree anatomy and haemodynamics between patients with and without stenoses of different degrees. Our goal is to elucidate the role of coronary anatomy and its dominating biomechanical stresses in the development of atherosclerotic plaques, with a particular focus on the initial stage of plaque onset. The novelty of the present work lies in several aspects:

- We compared patient-specific characteristics respectively at the whole-tree, bifurcating and non-bifurcating segment level, compared to existing work which focused mainly on the diseased branches or sectors,
- We considered all established adverse haemodynamic thresholds, each quantified in a relative manner, to eliminate the potential uncertainties around threshold choices, thereby benchmarking adverse haemodynamic characterization, and
- We conducted a joint investigation of both anatomy and the blood flow-induced biomechanical stresses in the course of plaque development, with a focus on their diagnostic capability in detecting plaque onset.

This work will contribute to the emerging effort of preventative diagnostic imaging for CAD [29], providing early warnings of risk prior to clinical plaque onset and progression through patient-specific quantitative coronary anatomy and biomechanical stress characterization.

## 2. Material and methods

### 2.1. Study population and coronary segmentation

The ASOCA dataset [30] comprises 40 CAD cases, from which we excluded one diseased case due to extreme stenosis in the left anterior descending (LAD) artery. Of the 39 left main coronary trees studied, 20 had no significant stenosis, 12 were moderately stenosed and 7 had severe stenoses.

This dataset is based on Computed Tomography Coronary Angiogram (CTCA) images obtained using a GE LightSpeed 64 slice CT Scanner with an ECG-gated retrospective acquisition protocol. The in-plane resolution of the acquired images was 0.3–0.4 mm, and the out-of-plane resolution was 0.625 mm [31]. All images were annotated by three experts independently using three-dimensional Slicer [32], followed by an automated majority voting method to derive the final vascular mask. Refer to Gharleghi *et al*. [30] for a detailed description of the process and table 1 for the patient demographics.

**Table 1.** Patient demographics and number of patients in each stenosis category. DS, diameter stenosis.

group	no stenosis (%DS = 0)	moderate stenosis (0 < %DS < 70)	severe stenosis (%DS ≥ 70)	*p*‐value
patients	20	12	7	
female	12 (60%)	2 (17%)	1 (14%)	0.039
male	8 (17%)	10 (83%)	6 (86%)	0.039
age	55 ± 8	57 ± 10	59 ± 11	0.0328

*p*-values were results of Chi-square tests for categorical variables or Welch’s *t* tests for continuous variables. Values in the parentheses represent the percentage of the values in the corresponding category

DS, diameter stenosis.

### 2.2. Computational model and boundary conditions

**Figure 1.**
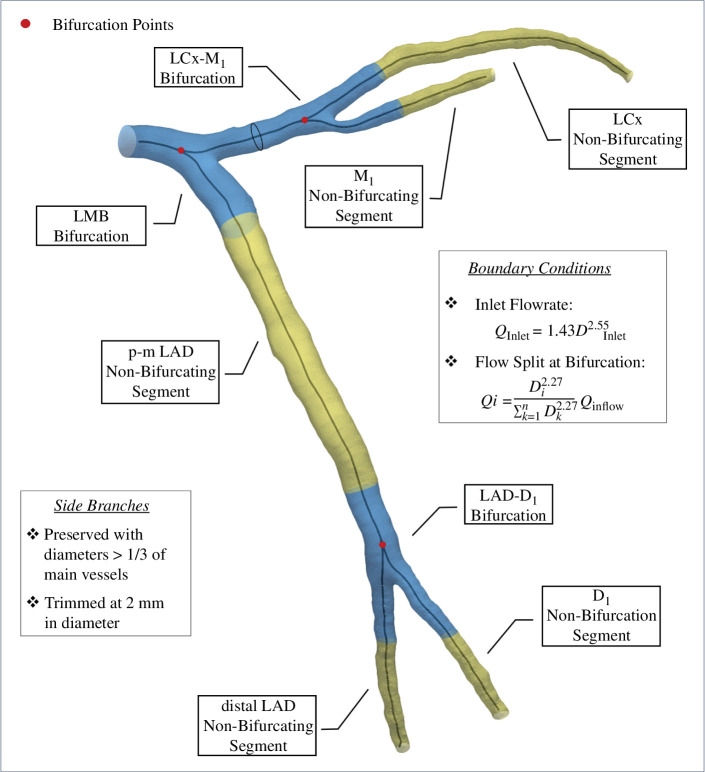
Computational settings and schematic of dissecting a left coronary artery tree into bifurcating (blue) and non-bifurcating (yellow) segments for sub-group analysis, with a bifurcation defined as 10 mm proximal and distal to a bifurcation point (red points) along the vascular centreline.

All distal coronary branches were trimmed if < 2 mm in diameter due to the limited resolution of CTCA. Only side branches with diameters more than one-third of the main vessels were preserved. Bifurcating and non-bifurcating coronary segments were defined as 10 mm centreline length proximal and distal to the bifurcation point following our previous study to allow cross-study comparison [31] (figure 1) and also to ensure no stenosis near a bifurcation was bisected, resulting in 53 bifurcations and 57 non-bifurcating segments for the 20 no-stenosis cases, and 54 bifurcations (12 with stenoses) and 62 non-bifurcating segments (14 with stenoses) for the 19 moderately or severely stenosed cases. (table 2) To examine the anatomical and haemodynamic differences by plaque severity, we used a percent diameter stenosis (%DS) to classify non-stenosed (%DS = 0), moderate (0 < %DS < 70) and severe stenosis (%DS ≥ 70).

**Table 2.** Breakdown of the stenosed and stenosis-free segments across the bifurcating and non-bifurcating groups. LMB: Left Main Bifurcation, LAD: Left Anterior Descending artery, LCx: Left Circumflex artery, D1: the 1st Diagonal artery, M1: the 1st Marginal artery, and p-m LAD indicates the proximal and middle segments of the LAD.

	group	coronary trees with no stenosis (%DS = 0)	coronary trees with moderate stenosis (30 < %DS < 70)		coronary trees with severe stenosis (%DS ≥ 70)	
			stenosis-free segments	stenosed segments	stenosis-free segments	stenosed segments
bifurcating segments	LMB	20	11	2	8	0
	LAD-D_1_	16	10	4	7	1
	LAD-D_2_	4	5	2	4	2
	LCx-M_1_	13	5	0	4	1
	total	53	31	14	23	4
non-bifurcating segments	LAD	19	11	2	7	2
	LCx	20	12	4	7	1
	DI	6	4	1	3	1
	LM	3	2	1	1	0
	M_1_	9	8	1	7	1
	total	57	37	9	25	5

Since patient-specific coronary flow conditions were unavailable, we adopted a standard uniform velocity profile and waveform from the literature [33] as the inlet condition after scaling according to the patient-specific inlet diameter *D*, which is reported to fit very well (*r* ^2 ^= 0.87) with intravascular Doppler measurement [34], deriving the scaled cycle-averaged flowrate *Q*:


(2.1) $$Q=1.43D^{2.55}.$$

The coronary haemodynamics were quantified assuming a resting state, with a flow split outflow strategy applied at each bifurcation, dividing the flowrate at the proximal main vessel into the daughter branches until all outlets were reached [34]:


(2.2) $$Q_{i}=\frac{D_{i}^{2.27}}{\sum_{k=1}^{n}D_{k}^{2.27}}Q_{\text{inflow}}.$$

where *n* is the number of daughter arteries at a bifurcation, *i* refers to the *i*th daughter branch and *D* is the diameter averaged from the parameterized centreline points along its 10 mm length, as recommended as appropriate for atherosclerotic arteries where *in vivo* data are unavailable [35].

Computational meshes of each coronary model were generated using ICEM-CFD embedded in the ANSYS package (v. 2023R1, Canonsburg, USA), with the maximal sizes of the surface and volume elements determined as 0.1 and 0.2 mm, following a mesh sensitivity analysis. The coronary wall was assumed to be rigid, with five prismatic boundary layers adhering to it to better resolve the near-wall blood flow. A laminar fluid model was used since the Reynolds number at the most stenosed region was below 2000. The blood flow was modelled as incompressible, and the Carreau-Yasuda non-Newtonian fluid model [36] was used to reflect the shear-thinning behaviour of blood:


(2.3) $$\mu =\mu_{i}+\frac{\mu_{0}-\mu_{i}}{{\left[1+{\left(\lambda \left|\overset{˙}{\gamma }\right|\right)}^{b}\right]}^{a}},$$

where μ is the viscosity, $\mu_{i}$ = 0.0035 Pa s is the high shear viscosity, $\mu_{0}$ = 0.16 Pa s is the low shear viscosity, λ = 8.2 s is the time constant and *a* = 0.64, *b* = 1.23, following Razavi *et al*. [36].

### 2.3. Coronary anatomy and haemodynamic analysis

An automated coronary shape analysis was performed on the three-dimensional geometries with the centrelines calculated using an in-house code based on the Vascular Modelling ToolKit (VMTK, v. 1.4). For all branches, we calculated the average absolute curvature *κ*_a_ to quantify vessel tortuosity, as recommended in recent literature [37], following


(2.4) $$\kappa_{a}=\frac{1}{L}\int_{s1}^{s2}\frac{\left|c^{\prime }\left(s\right)\times c^{\prime \prime }\left(s\right)\right|}{{\left|c^{\prime }\left(s\right)\right|}^{3}}ds,$$

where *c*(*s*) denotes the centreline parameterized along the coordinate *s* of curve *c*, and *L* represents the length of a curve considered. Furthermore, we calculated the mean maximal inscribed sphere diameter (MISD), and torsion *τ*_a_:


(2.5) $$\tau_{a}=\frac{1}{L}\int_{s1}^{s2}\frac{\left|c^{\prime }\left(s\right)\times c^{\;\prime \prime }\left(s\right)\right|\cdot c^{\;\prime \prime \prime }\left(s\right)}{{\left|c^{\prime }\left(s\right)\times c^{\;\prime \prime }\left(s\right)\right|}^{2}}ds.$$

For bifurcating segments, we additionally computed the inflow angle, bifurcation angle and the Finet’s ratio [31]:


(2.6) $$\text{FR}=\frac{D_{\text{PMV}}}{D_{\text{DMV}}+D_{\text{SB}}}.$$

A detailed definition of all considered anatomical metrics is given in table 3.

**Table 3.** Definitions of coronary geometric parameters for bifurcating and non-bifurcating segments.

	geometric parameters	definitions
non-bifurcating segments	MISD	mean diameter of the largest spheres that fit inside the coronary lumen at the points parameterized along the centreline
	absolute curvature	mean $\varkappa_{a}$ corresponding to the points parameterized along the centreline
	torsion	mean $\tau_{a}$ corresponding to the points parameterized along the centreline
bifurcating segments	inflow angle	angle with which a PMV enters the bifurcation plane, i.e. a least square plane fitted to all the *c*
	PMV/DMV/SB diameter	angle of a bifurcation between the DMV and the SB
	PMV/DMV/SB curvature	mean diameter of the PMV, DMV or SB
	PMV/DMV/SB torsion	mean absolute curvature of the PMV, DMV or SB
	Finet’s ratio	ratio of the mean PMV diameter to that of the mean DMV and SB diameters

DVM, Daughter Main Vessel; MISD, Maximal inscribed sphere diameter; PMV, Parent main vessel; SB, Side Branch.

Transient CFD simulations were performed using ANSYS-CFX for four cardiac cycles, with results taken from the fourth cycle to minimize transient start-up effects. A time step of 0.005 s was specified for the implicit second-order temporal discretization scheme. The criterion for convergence was set as 10^−4^ for the continuity and normalized velocity and pressure. We quantified the relative luminal area exposed to adversely low TAESS (%LowTAESS), high OSI (%HighOSI) and RRT (%HighRRT) due to their association with endothelial cell dysfunction and plaque development [26], calculated as:


(2.7) $$TAESS=\frac{1}{T}\int_{0}^{T}\left|\tau_{\omega }\right|dt,$$

(2.8) $$OSI=\frac{1}{2}\left(1-\frac{\left|\int_{0}^{T}\vec{\tau_{\omega }}dt\right|}{\int_{0}^{T}\left|\vec{\tau_{\omega }}\right|dt}\right),$$

(2.9) $$RRT=\frac{1}{\left(1-2\times OSI\right)\times TAESS},$$

where *τ_w_* is the flow-induced shear stress vector at the luminal wall, and *T* denotes the cardiac cycle period. In addition, the Topological Shear Variation Index (TSVI) that characterizes the endothelial contraction and expansion was calculated due to recent clinical evidence in predicting myocardial ischaemia [16], following


(2.10) $$TSVI\;=\;{\left[\frac{1}{T}\int_{0}^{T}{\left(DIV_{ESS}-\bar{DIV_{ESS}}\right)}^{2}dt\right]}^{1/2},$$

where DIV_ESS_ is the divergence of the ESS unit vector field, following the definition by Mazzi *et al*. [38].

It is important to note that although low TAESS, high OSI and RRT are generally considered to have adverse effects on the endothelial cells [26], different thresholds for each parameter have been proposed in the literature [22,27,39,40], with uncertainty around their pathophysiological relevance. Thus, for comparisons between non-stenosed (%DS = 0) and moderately or severely stenosed (%DS > 0) coronaries, we investigated and reported on all recommended thresholds for %LowTAESS, including 0.4, 0.5, 1.3 and 2.5 Pa, denoted as %LowTAESS@0.5 Pa, for example. Similarly, for %HighOSI, we studied thresholds of 0.1 and 0.2 [27,39]. Since RRT is derived from TAESS and OSI, eight thresholds emerged for %HighRRT, i.e. 0.50, 0.67, 0.96, 1.28, 2.50, 3.13, 3.33 and 4.17 Pa^−1^. We found consistent relationships regardless of the threshold chosen, as presented in the results (and detailed in electronic supplementary material, appendix A). Consequently, for the comparisons between moderately (0 < %DS < 70) and severely (%DS ≥ 70) stenosed coronaries, we focused only on the most used thresholds, i.e. %LowTAESS@0.5 Pa, %HighOSI@0.1 and %HighRRT@2.5 Pa^−1^.

### 2.4. Statistical analysis

The statistical analyses were conducted using the R language-based software JASP (v. 0.17.3). Continuous variables were expressed as mean and Standard Deviation (s.d.), and categorical variables were given as counts and percentages. Anatomic and haemodynamic differences between the non-, moderately and severely stenosed arteries were studied for the whole coronary trees, and all bifurcation and non-bifurcating segments. We used a Shapiro–Wilk test to check for the normality of all distributions before using a Welch’s *t*‐test for the normally distributed variables or a Mann-Whitney *U*-test for non-normally distributed variables; both are considered suitable for the comparison of small samples. To account for the multiple comparisons considered here, we adjusted the *p* values with a Bonferroni correction to reduce the chances of a false-positive result (type-I error) before interpreting their significance [41], whereby, after the correction, a *p* < 0.05 was considered statistically significant. A receiver operating characteristics (ROC) curve was used to reveal the diagnostic performance if a parameter was significantly different between groups with a minimal sample size >26, following a power estimation. The number of patients considered allowed us to only measure the sensitivity, specificity and area under the ROC curve (AUC) of a parameter in discriminating moderate or severe stenosed arteries (%DS > 0) against non-stenosed arteries (%DS = 0), but not moderately (0 <%DS <70) against severely stenosed arteries (%DS ≥ 70). Correlations between the anatomical and haemodynamic metrics were examined using the Pearson correlation coefficient *r*.

## 3. Results

Out of all metrics investigated for differences between non-stenosed (%DS = 0) and stenosed (%DS > 0) cases, only TSVI significantly differed across all three levels of comparison: the whole coronary tree, bifurcating and non-bifurcating segments (*p* < 0.002). The average absolute curvature and %HighOSI (although with small absolute values) differed significantly only for the whole trees (*p* < 0.024) and non-bifurcating segments (*p* < 0.027), whilst the side-branch diameter and distal main vessel torsion differed significantly for the bifurcating segments (*p* < 0.041).

**Figure 2.**
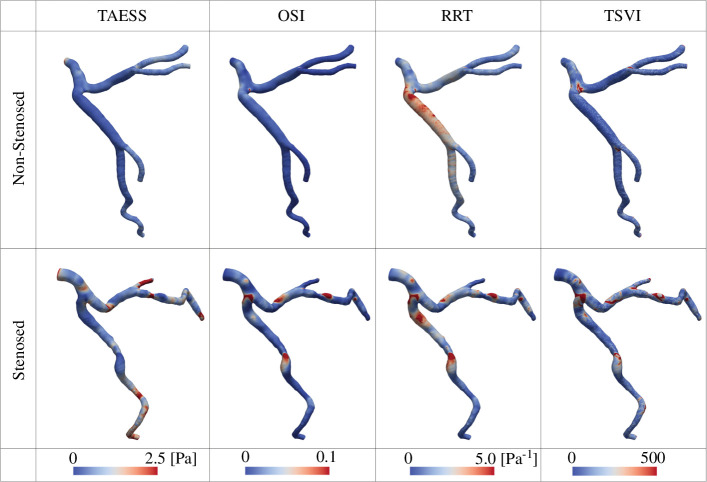
Time-averaged endothelial shear stress (TAESS), oscillatory shear index (OSI), relative residence time (RRT) and topological shear variation index (TSVI) plots from haemodynamic simulations of a representative non-stenosed and stenosed left coronary artery tree, where focal stenosis with %DS = 50 is located at the middle left circumflex artery. Major branches in the stenosed cases exhibit larger curvatures, resulting in higher TAESS, OSI, RRT and TSVI distributions across the entire coronary tree.

The choice of different thresholds to determine adverse haemodynamics (electronic supplementary material, appendix A), or excluding the severely stenosed cases to compare only the non-stenosed and moderately stenosed cases (%DS = 0 *vs.* 0 <%DS < 70, electronic supplementary material, appendix C), did not affect the statistical significance of these results. Stenosed coronary trees tend to be more curved than the non-stenosed, resulting in larger TAESS, OSI, RRT and TSVI distributions across the entire artery tree, rather than just within the stenosed segments (figure 2). When comparing moderately and severely stenosed arteries (0 < %DS < 70 versus %DS ≥ 70), no anatomical metrics showed statistical difference, and %LowTAESS and %HighRRT were the only haemodynamic parameters that significantly differed (*p* < 0.012).

### 3.1. Comparisons between non-stenosed and moderately or severely stenosed cases

**Figure 3.**
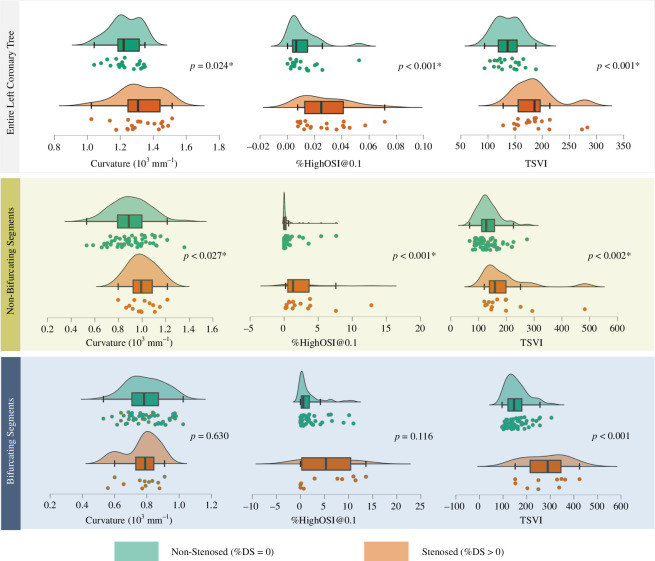
Comparisons of the average curvature, relative area exposed to high oscillatory shear index at threshold 0.1 (%HighOSI@0.1) and topological shear variation index (TSVI) between the non-stenosed (%DS = 0) and moderately or severely stenosed (%DS > 0) entire left coronary trees (Top: 20 versus 19), non-bifurcating (Middle: 57 versus 14) and bifurcating (Bottom: 53 versus 12) segments. Note: %HighOSI@0.1 is shown here as an example, with distributions of %HighOSI@0.2 presented in electronic supplementary material, appendix A and also demonstrating statistical difference between the non-stenosed and moderately or severely stenosed cases.

**Figure 4.**
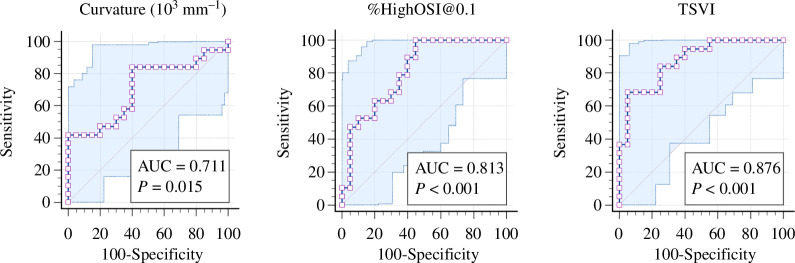
Diagnostic performance of the average curvature, relative area exposed to high oscillatory shear index at threshold 0.1 (%HighOSI@0.1) and topological shear variation index (TSVI) in differentiating between non-stenosed (%DS = 0) and moderately or severely stenosed (%DS > 0) left coronary arteries.

TSVI was the only parameter significantly higher in the stenosed coronaries across all three levels of comparison: the whole tree (186 ± 40 versus 137 ± 25, *p* < 0.001), bifurcating segments (282 ± 85 versus 157 ± 47, *p* < 0.001) and non-bifurcating segments (196 ± 96 versus 134 ± 40, *p* = 0.002), compared to the non-stenosed (figure 3). TSVI demonstrated the best classification performance in differentiating stenosed coronary arteries (%DS > 0), in terms of the highest AUC of 0.876 (95% CI: 0.713–0.960) at the optimal cut-off of 169 (*p* < 0.001, figure 4), with a sensitivity of 0.68 (95% confidence interval [CI]: 0.431–0.872) and a specificity of 0.92 (95% CI: 0.752–0.999).

Absolute average curvature was the only anatomical parameter significantly higher in the stenosed whole trees (1.321 ± 0.131 versus 1.230 ± 0.090 m^−1^, *p* = 0.024) and non-bifurcating segments (1.001 ± 0.116 versus 0.902 ± 0.162 m^−1^, *p* = 0.027), but not in the bifurcating segments (*p* > 0.057, figure 3). This is likely due to the complex interplay between different anatomical features around a bifurcation, such as the side-branch diameter (*p* = 0.041) and distal main vessel torsion (*p* = 0.024), which together affect the adverse ESS distribution and thus warrant further analysis to confirm their roles. Curvature had a high sensitivity of 0.842 (95% CI: 0.604–0.966) in identifying stenosed coronary arteries (%DS > 0) at the optimal cut-off of 1.227 m^−1^ (*p* = 0.015, figure 4), with a specificity of 0.600 (95% CI: 0.361–0.809) and an AUC of 0.711 (95% CI: 0.543–0.844). Within all anatomical features, coronary curvature negatively correlated with diameter (*r* = −0.474 and *p* = 0.002), suggesting that smaller arteries tend to be more curved than larger ones.

Similarly, %HighOSI, regardless of the thresholds chosen (@0.1 or @0.2), was statistically higher in the stenosed coronaries (%DS>70) than in non-stenosed coronaries (%DS = 0) for the entire coronary tree (0.028 ± 0.018 versus 0.011 ± 0.012, *p* < 0.001) and non-bifurcating segments (2.710 ± 3.438 versus 0.821 ± 2.004, *p* < 0.001), but not for the bifurcating segments (*p* > 0.116, figure 3). In detecting stenosed (%DS > 0) coronary trees, %HighOSI@0.1 had the highest sensitivity 0.999 (95% CI: 0.824–0.999) at the optimal cut-off of 0.006 (*p* < 0.001, figure 4), with an AUC of 0.813 (95% CI: 0.656–0.920) and a specificity of 0.550 (95% CI 0.315–0.769). However, OSI should be interpreted cautiously since the absolute values were generally very small (<0.4 in this study and the luminal area affected by adverse OSI was <15%).

### 3.2. Comparisons between moderately and severely stenosed cases

**Figure 5.**
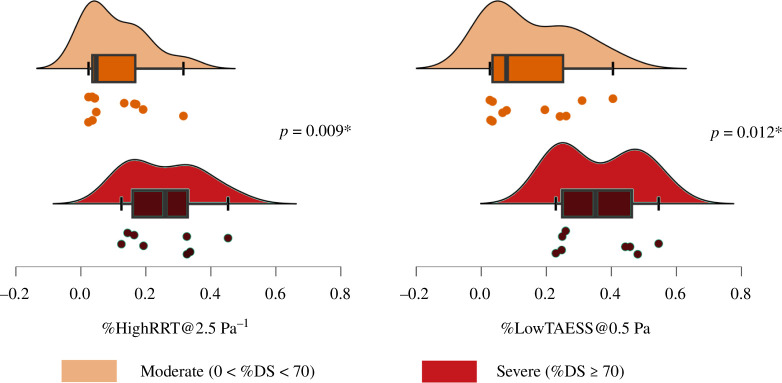
Comparisons of the relative areas subject to high relative residence time at threshold 2.5 Pa^−1^ (%HighRRT@2.5 Pa^−1^) and low time-averaged endothelial shear stress at 0.5 Pa (%LowTAESS@0.5 Pa) between moderately (0 < %DS<70, *n* = 12) and severely (%DS≥70, *n* = 7) stenosed coronary arteries.

When comparing only the stenosed cases, we found a statistically larger %LowTAESS@0.5 Pa (*p* = 0.009) in severely stenosed coronary trees (%DS ≥ 70: 0.364 ± 0.130) than in moderately stenosed trees (0 < %DS < 70: 0.153 ± 0.135). A similar trend was observed in %HighRRT@2.5 Pa^−1^ (moderate: 0.108 ± 0.095 versus severe: 0.258 ± 0.118, *p* = 0.012, figure 5). Neither TSVI (*p* = 0.114) nor %HighOSI (*p* = 0.665) showed significant differences, suggesting that both low and oscillatory shear stress play a role in the transition from moderate plaques to severe stenosis, with low ESS being a more important contributing factor due to the insignificant difference in %HighOSI. For anatomical metrics, none showed statistical difference across the coronary trees (*p* > 0.272), including the average absolute curvature. Segment-level comparison between the moderately and severely stenosed non-bifurcating and bifurcating segments was not performed, due to the limited sample size within the stenosed groups, i.e. only four severely stenosed bifurcations and five severely non-bifurcating segments in total.

## 4. Discussion

We systematically compared differences in the coronary anatomy and blood flow between non-, moderately and severely stenosed coronaries using our previously published dataset (ASOCA) of patients with suspected CAD. This is the first study to assess a comprehensive set of anatomical and haemodynamic factors with established clinical relevance across the whole left coronary trees at different disease stages. Previous works either looked only at the disease affected vessels [10] or considered only steady-state haemodynamic factors without account for changes in blood flow over a cardiac cycle [42]. Our results on both entire coronary tree and the dissected segments highlight the potential of coronary anatomy and blood flow characteristics to be used for coronary plaque onset and progression prediction.

The initial presentation of atherosclerotic plaques involves coronary wall thickening outwards, known as extensive compensatory remodelling (or positive remodelling). Plaques at this stage have no lumen-protruding effects [43], making them difficult to detect by invasive coronary angiography. Consequently, key factors driving atherogenesis and vascular injury, including coronary arterial anatomy and its local haemodynamics, are being extensively studied to alarm plaque formation risks. Beyond the conventional interests in low and oscillatory shear theory, emerging efforts aim to elucidate the impacts of shear stress multi-directionality and topological skeleton [16,17,20,44,45].

Recent works include notably a detailed comparison of a full set of ESS-derived metrics simulated by fluid–structure interaction [17] and a prognostic efficacy study for TSVI to predict myocardial infarction in CAD patients [16]. For closer relevance to clinical applications and considering that the CTCA images were reconstructed only at the end-diastolic phase, we focused only on clinically established haemodynamic factors, including low and oscillatory shear stress and TSVI, assuming rigid wall and no dynamic bending of the coronary vessels. Unlike conventional ESS-derived metrics, TSVI identifies the regions exposed to significant ESS divergence, characterizing the degree of vascular wall contraction and expansion driven by blood flow. TSVI proved to be robust differentiator of stenosed coronaries, which is the only parameter exhibiting statistically significant difference at all comparison levels including the whole tree, bifurcating and non-bifurcating segments. This indicates its potential for plaque risk assessment, particularly given that coronary bifurcations are predisposed to plaque formation. However, longitudinal studies from the healthy state are needed to establish pathologically relevant thresholds and verify their predictive capability.

While TAESS and OSI have both been considered drivers of plaque onset [26], we observed only OSI to differ statistically between the non-stenosed and stenosed left coronary arteries. OSI was first linked to atherosclerosis in postmortem human carotid arteries [11], and later correlated with intimal thickness and suggested for coronary arteries as well [13,46]. The underlying mechanism may involve OSI increasing smooth muscle cell proliferation and migration [47], which explains our findings here. A prior study found equivalent efficacy for TAESS and OSI in predicting plaque onset, with TAESS predicting plaque locations significantly and OSI and RRT having fewer false negative predictions [24,25]. However, this study used diseased patient-specific coronaries with plaques virtually removed to model the healthy state, which may introduce great uncertainties, as arterial anatomy could have altered as a result of disease development, and virtual removal of plaques from stenosed arteries can under- or overestimate the size of the healthy lumen [26]. In our study, we kept the carefully annotated coronary arteries intact, and analysed only the relative areas exposed to established adverse haemodynamic thresholds to minimize the potential impact from local stenoses on global haemodynamic metrics quantification. In another longitudinal study on human abdominal aortas, early atherosclerotic lesions co-localized with both low TAESS and high OSI [48]. However, such findings in the context of other vessels may not hold true for coronaries, as blood flow in the aorta has much greater Reynolds numbers and thus distinct flow regimes.

Among these haemodynamic parameters, TAESS estimates the temporal average of the ESS magnitudes, while OSI characterizes the degree of shear stress deviations from the mean shear vector, which has often been misinterpreted as reflecting the degree of flow reversal [44], and RRT identifies regions exposed to both low and oscillatory ESS. OSI and RRT may thus more comprehensively reflect the variation of both shear stress magnitude and direction on endothelial cells in a cardiac cycle. However, caution is warranted in interpretation, since OSI is typically very low in coronaries, compared to carotid and aorta. In addition, fundamental studies on how OSI or RRT affect endothelial cells are lacking, in contrast to ESS, whereby low TAESS, proximal or distal to a focal plaque, has been linked to plaque progression, and high TAESS at the plaque spot promote plaque erosion [10].

Following similar protocols, our findings of OSI difference before and after plaque onset, and RRT difference between the moderate (0 < %DS < 70) and severe (%DS ≥ 70) stenoses, warrant further observations on large-scale longitudinal cohorts. This is particularly relevant given that a recent animal experiment has endorsed the effects of RRT [20,49]. The insignificant differences for bifurcating segments, where complex flow disturbances are governed by a multitude of anatomical factors, call for additional observation to examine the underlying confounding effects. In addition to factors with established links to endothelial dysfunction, future work should include flow characteristics that are known to prevent plaque formation. One such characteristic is the intensity of helical flow [50,51], which has shown protective effects in animal studies [52].

For vascular anatomy, the link to plaque onset and progression remains inconclusive. Various studies have suggested vascular tortuosity to play a role [53,54]. However, its definition has been inconsistent, and some metrics, e.g. the tortuosity index, are incapable of capturing the actual bending of three-dimensional vessels [37]. Clinical studies typically measure tortuosity on two-dimensional X-ray fluoroscopy images, defining it by the number of arterial bending with angles over a certain degree, e.g. 90° [6], or by classifying the shape of vessels concerning their two-dimensional projections, e.g. C- or S-shaped [55]. Even when tortuosity was calculated by engineers on three-dimensional centrelines, various equations have been used, including the tortuosity index [56] and average absolute curvature [57], etc. The inconsistencies in measuring tortuosity have thus hindered cross-comparison of different studies. Moreover, tortuosity may be affected by variations in the coronary diameter [58], suggesting potentially confounding effects on the local haemodynamics and, therefore, warrants further detailed analysis. Besides these centreline-based anatomical metrics, recent studies tend to test anatomical metrics based on the length or volume of the coronary arteries, e.g. the coronary artery volume index [59], whereby a high prognostic efficacy for cardiovascular events was reported.

The absence of patient-specific boundary conditions for our flow simulations may be considered a limitation. However, coronary flow measurement is with great uncertainty and not routinely performed in cardiological practice, and thus may not be feasible for analysis and comparison of large retrospective registries. Non-invasive measurement of coronary artery flow relies on transthoracic Doppler echocardiography, whereas the depth and resolution of imaging, obstruction of the bones, etc. have constrained its use within the arteries of LM, LAD and PDA. Invasive approaches capable of measuring the side-branch flows include Doppler sonography performed during Intravascular Ultrasound (IVUS) and TIMI Frame Count (TFC) that derives flow velocity from the counts of cine-angiographic frames [60]. Here, we adopted a scaling method (*n* = 2.55) to estimate the left coronary inflow based on the average diameter of the Left Main (LM) artery, which correlates well with IVUS-obtained flowrates (*r* ^2^ = 0.87) [34] and is the most common inflow strategy in this scenario with normalized ESS characterized in good agreement with that by boundary conditions measured *in vivo* [35]. Thus, standardizing the inflow assumption using the flow-diameter relation for population groups improves the simulation efficiency and assures physiologically realistic results.

For outflow conditions, a lumped parameter model is usually applied to account for the stenosis-induced flow redistribution within the epicardial arteries [61]. This approach requires the total myocardial resistance to be derived from the volume of the heart muscle and is commonly used to capture the fractional flow reserve under the hyperaemic condition [62]. To facilitate the characterization of flows for a large population, we previously compared the WSS and pressure drop between coronaries with and without stenosis, respectively under the resting and hyperaemic condition [63]. The results suggested a mild difference in the average ESS and pressure drop across stenosis of different severities, supporting a flow split method to be used at bifurcations under the resting condition.

This study has limitations. Although we used the largest open-source CAD dataset, the sample size is still relatively small due to its retrospective and single-centre nature, where the comparisons between small samples were accounted for by using the Welch’s *t* tests rather than the student *t* tests. Moreover, we have only included the most used haemodynamic metrics with established links to endothelial dysfunction. Future work would benefit from incorporating recently proposed novel parameters, e.g. the cross flow index and transverse ESS [17], to better account for the multidirectional nature of coronary blood flow patterns.

In terms of the simulation methods, we assumed a rigid wall and no dynamic bending of the coronaries, as only end-diastolic CTCA images were available, preventing a patient-specific characterization of the cardiac motion. However, recent studies have highlighted the effects of coronary dynamics on OSI quantification, especially for arteries with smaller stenosis degrees [17]. Our findings warrant further verification against fluid–structure interaction simulations, and future study on earlier stages of atherosclerosis development is recommended to consider the effects of heart motion [44].

Finally, we have included only symptomatic patients with no, moderate or severe stenoses at their first visit, without follow-ups to confirm their longitudinal changes. An ideal cohort for this study would include patients having both follow-up images and baseline images prior to their plaque onset or progression. We had not manually removed the plaques to create a virtually healthy artery as performed in prior studies [24,25], as the uncertainties introduced in this procedure would be challenging to assess [26]. Instead, we quantified the relative vascular areas exposed to adverse haemodynamic at different thresholds to minimize the potential impact of the focal stenoses on global haemodynamic metrics quantification. However, we acknowledge the critical importance of longitudinal studies starting from the healthy state to understand better the causal effects of coronary shape and blood flow on plaque development.

## 5. Conclusion

Through comparing anatomical and blood flow features of the whole left coronary tree, and the dissected bifurcating and non-bifurcating segments, we found that only TSVI statistically differed between non-stenosed (%DS = 0) and stenosed (%DS > 0) cases across the three comparison levels (*p* < 0.002) with the best diagnostic performance (AUC = 0.876), whereas curvature and %HighOSI differed only for the whole tree (*p* < 0.024) and non-bifurcating segments (*p* < 0.027), with inferior diagnostic performance (AUC > 0.711). The previously recognized metrics %LowTAESS or %HighRRT only statistically differed between coronaries with moderate (0 < %DS < 70) and severe (%DS ≥ 70) stenoses (*p* < 0 .012). This suggests that TSVI, coronary curvature and OSI may promote plaque onset, while TAESS and RRT play a role in the progression of plaques from the moderate to severe disease stage. Our findings contribute to a clearer understanding of the anatomical and haemodynamic drivers of atherosclerotic plaque initiation and progression, which are directly relevant to the emerging efforts of preventative diagnostic imaging of CAD.

## Data Availability

The medical images used to reconstruct the patient coronary geometries have been made publicly available through the UK Data Service [64]. Detailed anatomical analysis and haemodynamic simulation results have been provided in the supplementary material [65].
